# Risk of COVID-19 diagnosis and death in patients with mental illness: a cohort study

**DOI:** 10.1017/S2045796021000597

**Published:** 2021-10-14

**Authors:** Jeon-Yeon Seon, Sunjea Kim, Minha Hong, Min Kyoung Lim, In-Hwan Oh

**Affiliations:** 1 Health Insurance Research Institute, National Health Insurance Service, Wonju-si, Republic of Korea; 2 Department of Psychiatry, Myongji Hospital, Hanyang University, College of Medicine, Goyang-si, Republic of Korea; 3 Department of Preventive Medicine, Kyung Hee University School of Medicine, Seoul, Republic of Korea

**Keywords:** Bipolar disorder, chronic conditions, dementia, schizophrenia

## Abstract

**Aims:**

Patients with mental illness are vulnerable to severe acute respiratory syndrome coronavirus 2 (SARS-CoV-2) infection because of behavioural changes associated with cognitive deterioration, especially without their caregivers. While studies have reported that SARS-CoV-2 infection risk and severe clinical outcomes are high among patients with mental illness, there is a lack of quantitative research supporting this claim. This study investigates if SARS-CoV-2 infection and coronavirus disease 2019 (COVID-19)-related death are higher in patients with mental illness than among those without a mental disorder.

**Methods:**

A cohort study was conducted using the COVID-19 database of the National Health Insurance Service in South Korea. A total of 123 480 patients aged ⩾20 years who visited a hospital between 1 January 2020 and 30 May 2020 were analysed. Mental disorder diagnoses and types were determined based on 2019 medical records, and a multivariate logistic regression model was used to calculate the odds ratios (ORs) for SARS-CoV-2 infection and deaths.

**Results:**

The ORs for SARS-CoV-2 infection (OR 1.58; 95% CI 1.45–1.71) and COVID-19-related death (OR 2.18; 95% CI 1.57–3.04) were high among patients with mental illness. The OR of SARS-CoV-2 infection was higher among patients with severe mental illness (OR 2.60; 95% CI 2.21–3.06), dementia (OR 1.90; 95% CI 1.62–2.22) and substance use disorder (OR 4.98, 95% CI 3.60–6.88). The OR for COVID-19-related death was high among patients with severe mental illness (OR 3.53; 95% CI 1.82–6.83) and dementia (OR 2.12; 95% CI 1.39–3.22).

**Conclusions:**

Patients with mental illness are at high risk for SARS-CoV-2 infection and COVID-19-related death. Behavioural changes associated with cognitive deterioration and long-term care facility residence increase SARS-CoV-2 infection risk, and severe medical conditions and delayed treatment increase the COVID-19-related mortality risk in patients with mental illness. Patients with mental illness are a priority target population for COVID-19 prevention and treatment, and it is important to plan prevention measures that address their needs.

## Introduction

Coronavirus disease 2019 (COVID-19) is a novel infection caused by severe acute respiratory syndrome coronavirus 2 (SARS-CoV-2) (Wiersinga *et al*., 2020). After the World Health Organization (WHO) declared COVID-19 a pandemic on March 2020, 81 658 440 confirmed cases were reported worldwide in December 2020, and 1 802 206 of those led to death (WHO, 2020*a*). In South Korea, 61 769 confirmed cases and 917 deaths were reported in December 2020, indicating a mortality rate of approximately 1.5% (Ministry of Health and Welfare, 2020).

Personal infection prevention activities, such as mask-wearing, washing hands and social distancing, are important to reduce SARS-CoV-2 infection risk (Doung-Ngern *et al*., 2020; Shi *et al*., 2021). Patients with mental illness are vulnerable to SARS-CoV-2 infection because of behavioural changes associated with cognitive deterioration (Yao *et al*., 2020), especially without their caregivers. Some experts have proposed that patients with mental illness are at high risk of SARS-CoV-2 infection due to a lack of awareness of infection risk and a lack of commitment to personal prevention activities (Druss, 2020; Shinn and Viron, 2020; Yao *et al*., 2020).

Severe COVID-19 outcomes following SARS-CoV-2 infection are also a concern for patients with mental illness. Psychiatric symptoms are associated with health-risk behaviours such as smoking and drinking (Scott and Happell, 2011; Bartlem *et al*., 2015), explaining the higher prevalence of non-communicable diseases (NCDs) among patients with mental illness than in the general population (Himelhoch *et al*., 2004; Walker *et al*., 2015). The risk factors of severe COVID-19 identified to date are male sex, older age and severe medical conditions (Richardson *et al*., 2020; Williamson *et al*., 2020; Zhou *et al*., 2020). The high prevalence of NCDs and poor prognoses for subsequent medical conditions increase the risk of adverse outcomes following SARS-CoV-2 infection in patients with mental illness (Momen *et al*., 2020). While studies have reported that SARS-CoV-2 infection risk and severe clinical outcomes are high among patients with mental illness due to their clinical characteristics (Druss, 2020; Yao *et al*., 2020), there is a lack of quantitative research supporting this claim.

This study aimed to investigate whether SARS-CoV-2 infection risk and COVID-19-related death are higher among patients with mental illness than among those without a mental illness. We analysed the association between infection risk and mortality from SARS-CoV-2 and patients with mental illness, using data from a South Korean database.

## Material and methods

### Data collection and study participants

**Fig. 1.**
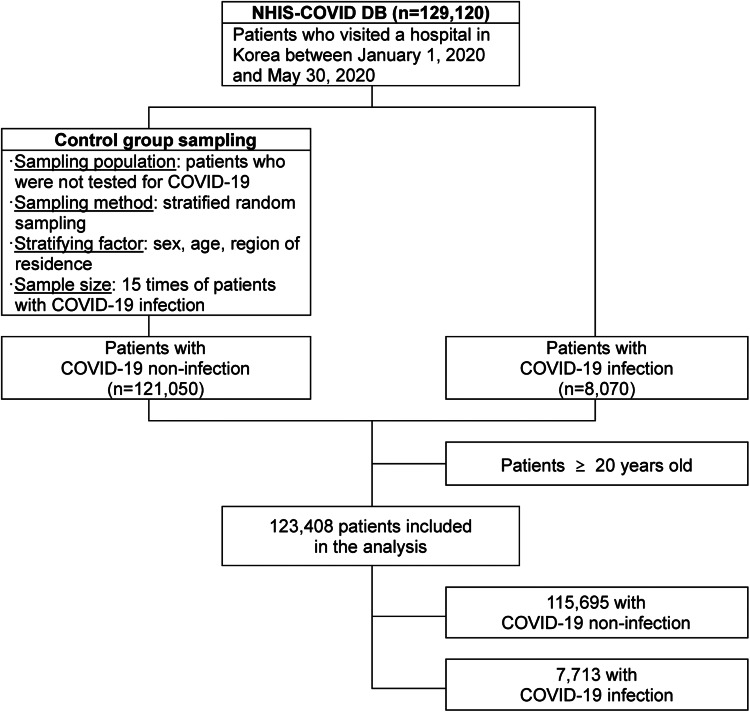
Flow chart for patients included in the analysis

Data from the COVID-19 database of National Health Insurance Service (NHIS-COVID DB), which combines data from the Korea Disease Control and Prevention Agency (KDCA) and the National Health Insurance Service (NHIS) were used to analyse the association between infection risk and mortality from SARS-CoV-2 in patients with mental illness. The NHIS-COVID DB provides nationwide data on eligibility, insurance, medical examination results and treatment history of COVID-19 and control groups (National Health Insurance Service, 2020). The database includes comprehensive 5-year medical records of 8070 COVID-19 patients diagnosed between 1 January 2020 and 30 May 2020, and of those in the control group who did not have a history of being tested for COVID-19 during that time. The control group was 15 times (121 050 patients) the size of the COVID-19 group. Patients from the control group without a history of SARS-CoV-2 testing were selected using stratified sampling. To stratify factors, sex (male, female), age (0–9, 10–19, 20–29, 30–39, 40–49, 50–59, 60–69, 70–79 and ⩾80 years) and region of residence (Seoul, Gyeonggi-do, Daegu, Gyeongsangbuk-do, others) were considered, and random sampling was used within a single stratum (National Health Insurance Service, 2020). Since paediatric and adolescent patients with a mental illness exhibit characteristics different from those of adult patients, children aged 19 years and under were excluded. In total, 123 408 patients aged ⩾20 years from the NHIS-COVID DB were included (Fig. 1).

In this study, a patient with mental illness was defined as a patient diagnosed with a mental illness as the primary diagnosis in 2019. The International Statistical Classification of Diseases (ICD-10) code corresponding to a mental illness is ‘F00–F99 (mental and behavioural disorders)’. Patients who visited an outpatient clinic at least three times for the same main diagnosis or were admitted to a hospital at least once were considered to have a mental illness. Patients with schizophrenia (F20–29) and bipolar disorder (F30, F31, F38, F39) were classified as having a severe mental illness. These patients were further divided into depression disorder (F32, F33, F34), dementia (F00, F01, F02, F03), substance use disorder (SUD) (F10–19), and anxiety and obsessive-compulsive disorder (F40, F41, F42) groups according to the type of their mental illness.

### Statistical analysis

We compared the characteristics of patients according to SARS-CoV-2 infection and COVID-19-related death using a χ^2^ test. Patient characteristics, current history, and type of mental illness, sex, age, region of residence, insurance cost and Charlson Comorbidity Index (CCI) scores were considered. Patients were categorised according to their age at 10-year intervals. For the region of residence, cities where a large number of confirmed cases had been reported, including Seoul, Daegu, Gyeonggi-do, Gyeongsang-buk-do and others, were considered. Since insurance costs are proportional to income in South Korea, insurance premiums were used as a marker of financial status. Medical aid recipients are low-income individuals who are exempt from health insurance payments. Patients who paid for health insurance were divided into the 1st (lowest), 2nd, 3rd, 4th and 5th (highest) quintiles according to their insurance costs. CCI scores represent the severity of comorbidities. Updated weights proposed by Quan *et al.* based on medical records from 2019 were applied to the scores (Quan *et al*., 2011).

Logistic regression analysis was performed to investigate the risk of SARS-CoV-2 infection and COVID-19-related deaths in patients with mental illness. The primary outcome was the SARS-CoV-2 infection. The odds ratios (ORs) were calculated using logistic regression analysis to analyse the risk of COVID-19 according to the mental illness diagnosis and type. Additionally, a multivariate logistic regression model was created with insurance premiums and CCI scores as independent variables to adjust the ORs. Since the model was adjusted for sex, age and region of residence during the control group sampling, these variables were not included as confounding variables.

The secondary outcome was COVID-19-related death. A multivariate logistic regression model adjusted for sex, age, the region of residence and insurance premiums; CCI score was used to calculate the OR for COVID-19 in patients with mental illness. SAS 9.4 was used for statistical analyses with the level of statistical significance set to *p*-value <0.05.

### Ethics statement

This study conformed to the Guidelines on De-identification of Personal Data of Korea and was approved by the Kyung Hee University's Institutional Review Board (IRB No. KHSIRB-20-301(EA)) as a review exemption study. As the study used de-identified data, the requirement for informed consent was waived by the board.

## Results

### Patient characteristics

To compare the risks of SARS-CoV-2 infection and COVID-19-related death according to patient characteristics, a *χ*^2^ test was performed with sex, age, the region of residence, health insurance premium, CCI score and mental illness as the independent variables (Table 1). Uninfected people in the NHIS-COVID DB were stratified 15-fold and sampled such that their sex, age and region of residence distributions matched those of those infected with SARS-CoV-2. This allowed for the equalisation of the sex, age and region of residence distributions between the infected and uninfected groups. However, significant differences in SARS-CoV-2 infection were found according to health insurance premiums, CCI scores and mental illness. People with SARS-CoV-2 infection were higher in the low-income group, and non-infected people were higher in the high-income group. In the case of high CCI and people with mental illness, column percentages of people with SARS-CoV-2 infection were higher. A *χ*^2^ test was used to assess the statistical significance of differences in COVID-19-related death rates according to sex, age, region of residence, health insurance premiums, CCI scores and mental illness (*p* < 0.001). As reported in Table 2, a *χ*^2^ test was performed to examine the association between the type of mental illness and SARS-CoV-2 infection risk, as well as the association between the type of mental illness and COVID-19-related mortality. Severe mental illnesses (schizophrenia and bipolar disorder), dementia, anxiety, obsessive-compulsive disorder and SUDs were found to be significantly associated with SARS-CoV-2 infection. Severe mental illness, depression and dementia were all significantly associated with COVID-19-related mortality, and the rate of COVID-19-related death was higher among patients with these disorders than among those without.

**Table 1.** Patients baseline characteristics

	Total (*N* = 123 408)				*p*-value^a^	COVID-19 patients (*N* = 7713)				*p*-value^a^
	Non COVID-19 patients (*n* = 115 695)		COVID-19 patients (*n* = 7713)			Survival (*n* = 7465)		Death (*n* = 248)		
	*n*	%^b^	*n*	%^b^		*n*	%^b^	*n*	%^b^	
Sex										
Male	45 720	39.5	3048	39.5	1.000	2912	39.0	136	54.8	<0.001
Female	69 975	60.5	4665	60.5		4553	61.0	112	45.2	
Age (years)										
20–29	30 855	26.7	2057	26.7	1.000	2057	27.6	0	0.0	<0.001
30–39	12 480	10.8	832	10.8		831	11.1	1	0.4	
40–49	15 540	13.4	1036	13.4		1032	13.8	4	1.6	
50–59	23 505	20.3	1567	20.3		1552	20.8	15	6.0	
60–69	17 985	15.5	1199	15.5		1161	15.6	38	15.3	
70–79	9255	8.0	617	8.0		551	7.4	66	26.6	
80+	6075	5.3	405	5.3		281	3.8	124	50.0	
Region of residence										
Seoul	7650	6.6	510	6.6	1.000	506	6.8	4	1.6	<0.001
Daegu	75 540	65.3	5036	65.3		4884	65.4	152	61.3	
Gyeonggi-do	6465	5.6	431	5.6		414	5.5	17	6.9	
Gyeongsangbuk-do	13 995	12.1	933	12.1		878	11.8	55	22.2	
Others	12 045	10.4	803	10.4		783	10.5	20	8.1	
Health insurance premium										
Medical Aid	6239	5.4	767	9.9	<0.001	718	9.6	49	19.8	<0.001
1st quintile (lowest)	22 072	19.1	1565	20.3		1528	20.5	37	14.9	
2nd quintile	17 510	15.1	1065	13.8		1045	14.0	20	8.1	
3rd quintile	19 761	17.1	1256	16.3		1224	16.4	32	12.9	
4th quintile	21 725	18.8	1261	16.3		1223	16.4	38	15.3	
5th quintile (highest)	28 388	24.5	1799	23.3		1727	23.1	72	29.0	
CCI score										
0	100 936	87.2	6625	85.9	0.001	6465	86.6	160	64.5	<0.001
1	6905	6.0	482	6.2		450	6.0	32	12.9	
2+	7854	6.8	606	7.9		550	7.4	56	22.6	
Mental illness										
No	109 219	94.4	6976	90.4	<0.001	6822	91.4	154	62.1	<0.001
Yes	6476	5.6	737	9.6		643	8.6	94	37.9	

COVID-19, Coronavirus Disease 2019; CCI, Charlson Comorbidity Index, Mental illness (F00–F99).

a *p*-value for *χ*^2^ test.

b % is column percentages

**Table 2.** Comparison of SARS-CoV-2 infection rate and COVID-19 mortality by types of mental illness

	Total (*N* = 123 408)				*p*-value^a^	Patients with COVID-19 infection (*N* = 7713)				*p*-value^a^
	COVID-19 non-infection (*n* = 115 695)		COVID-19 infection (*n* = 7713)			Survival (*n* = 7465)		Death (*n* = 248)		
	*n*	%^b^	*n*	%^b^		*n*	%^b^	*n*	%^b^	
Severe mental illness										
No	114 814	97.5	7523	97.5	<0.001	7292	97.7	231	93.1	<0.001
Yes	881	0.8	190	2.5		173	2.3	17	6.9	
Depression disorder										
No	113 865	98.4	7578	98.2	0.252	7339	98.3	239	96.4	0.022
Yes	1830	1.6	135	1.8		126	1.7	9	3.6	
Dementia										
No	114 330	98.8	7518	97.5	<0.001	7331	98.2	187	75.4	<0.001
Yes	1365	1.2	195	2.5		134	1.8	61	24.6	
Anxiety and OCD										
No	114 430	98.9	7610	98.7	0.049	7363	98.6	247	99.6	0.194
Yes	1265	1.1	103	1.3		102	1.4	1	0.4	
Substance use disorder										
No	115 570	99.9	7658	99.3	<0.001	7413	99.3	245	98.8	0.345
Yes	125	0.1	55	0.7		52	0.7	3	1.2	

COVID-19, Coronavirus Disease 2019; OCD, Obsessive-Compulsive disorder.

Severe mental illness (F20-F29, F30, F31, F38, F39), Depression disorder (F32, F33, F34), Dementia (F00, F01, F02, F03), Anxiety and obsessive-compulsive disorder (F40, F41, F42), Substance use disorder (F10–F19).

a *p*-value for *χ*^2^ test.

b % is column percentages.

### Analysis for risk of COVID-19 infection and death by mental illness severity

Logistic regression analysis was performed to analyse the risks of SARS-CoV-2 infection and COVID-19-related deaths in patients with mental illness (Table 3). Statistical significance in the risk of SARS-CoV-2 infection was found according to health insurance premiums, CCI scores and mental illness. Medical aids were less likely to be infected by SARS-CoV-2 compared to those with health insurance, and SARS-CoV-2 infection risk increased as CCI scores increased. No statistically significant difference in the SARS-CoV-2 infection risk was found between patients with CCI scores of 0 and those with CCI scores of 1. The OR for SARS-CoV-2 infection was high (1.10) for patients with CCI scores of ⩾2 (95% CI 1.01–1.20, *p*-value = 0.032), showing a high risk of SARS-CoV-2 infection. The OR for SARS-CoV-2 infection in patients with mental illness was 1.58 (95% CI 1.45–1.71, *p-*value < 0.001). Thus, patients with mental illness were found to be at a high risk of SARS-CoV-2 infection.

**Table 3.** Odds ratio for risk of SARS-CoV-2 infection and COVID-19 death

	OR for risk of COVID-19 infection (*n* = 123 480)			OR for risk of COVID-19 death (*n* = 7713)		
	Adjusted OR	95% CI	*p*-value	Adjusted OR	95% CI	*p*-value
Sex						
Male	–	–	–	1(ref)	1(ref)	1(ref)
Female	–	–	–	0.40	(0.30–0.54)	<0.001
Age (years)						
0–59	–	–	–	1(ref)	1(ref)	1(ref)
60–69	–	–	–	6.92	(3.97–12.08)	<0.001
70–79	–	–	–	27.08	(16.08–45.59)	<0.001
80+	–	–	–	97.62	(58.32–163.4)	<0.001
Region of residence						
Seoul	–	–	–	1(ref)	1(ref)	1(ref)
Daegu	–	–	–	2.27	(0.77–6.69)	0.138
Gyeonggi-do	–	–	–	4.12	(1.24–13.68)	0.021
Gyeongsangbuk-do	–	–	–	2.57	(0.84–7.83)	0.098
Others	–	–	–	2.59	(0.80–8.38)	0.113
Health insurance premium						
Medical aid	1(ref)	1(ref)	1(ref)	1(ref)	1(ref)	1(ref)
1st quintile (lowest)	0.63	(0.57–0.69)	<0.001	0.52	(0.31–0.85)	0.009
2nd quintile	0.54	(0.49–0.60)	<0.001	0.61	(0.34–1.11)	0.108
3rd quintile	0.57	(0.52–0.62)	<0.001	0.78	(0.46–1.31)	0.342
4th quintile	0.52	(0.47–0.57)	<0.001	0.56	(0.34–0.93)	0.024
5th quintile (highest)	0.56	(0.51–0.61)	<0.001	0.60	(0.39–0.94)	0.026
CCI score						
0	1(ref)	1(ref)	1(ref)	1(ref)	1(ref)	1(ref)
1	1.05	(0.95–1.15)	0.332	2.10	(1.35–3.28)	0.001
2+	1.10	(1.01–1.20)	0.032	1.26	(0.87–1.82)	0.231
Mental illness						
No	1(ref)	1(ref)	1(ref)	1(ref)	1(ref)	1(ref)
Yes	1.58	(1.45–1.71)	<0.001	2.18	(1.57–3.04)	<0.001

OR, Odds Ratio; CI, Confidence Interval; CCI, Charlson Comorbidity Index.

Mental illness (F00–F99).

Significant differences in COVID-19-related death rates were found according to sex, age, region of residence, health insurance premiums, CCI score and mental illness. The risk of COVID-19-related death was lower for women (OR 0.40, 95% CI 0.30–0.54, *p*-value < 0.001) and increased with age. A statistical difference in the risk of COVID-19-related death was found between Seoul and Gyeonggi-do (OR 4.12; 95% CI 1.24–13.68, *p*-value = 0.021). The risk of COVID-19-related death was higher in Gyeonggi-do than in Seoul. Among patients with health insurance, those in the 1st quintile (OR 0.52, 95% CI 0.31–0.85, *p*-value = 0.009), 4th quintile (OR 0.56, 95% CI 0.34–0.93, *p*-value = 0.024) and 5th quintile (OR 0.60, 95% CI 0.39–0.94, *p*-value = 0.026) showed significant differences in COVID-19-related mortality risk. The risk of COVID-19-related death was relatively low in patients with health insurance compared to those receiving medical aid. Patients with CCI scores of 1 (OR 2.10, 95% CI 1.35–3.28, *p*-value = 0.001) were at a higher risk of COVID-19 than those with CCI scores of 0. The OR for COVID-19-related death in patients with mental illness was high at 2.18 (95% CI 1.57–3.04, *p*-value < 0.001), showing that patients with mental illness had a higher risk of COVID-19-related death.

**Fig. 2.**
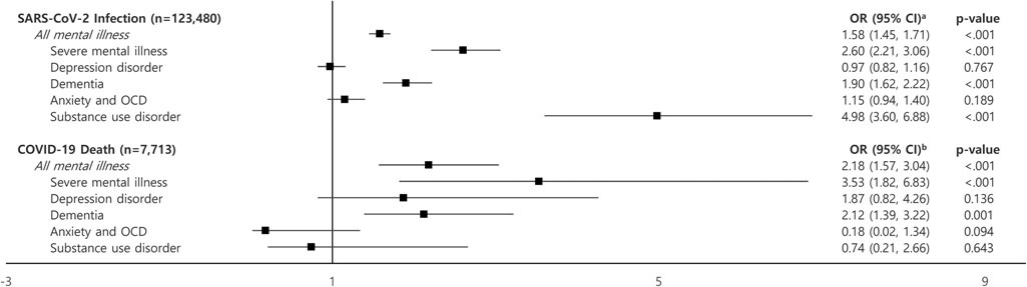
Odds ratio for risk of SARS-CoV-2 infection and COVID-19 death by types of mental illness. ^a^Adjusted for health insurance premium, Charlson Comorbidity Index score. ^b^Adjusted for sex, age, region of residence, health insurance premium, Charlson Comorbidity Index score. COVID-19, coronavirus disease 2019; OCD, obsessive-compulsive disorder; OR, odds ratio; CI, confidence interval. Severe mental illness (F20–F29, F30, F31, F38, F39), depression disorder (F32, F33, F34), dementia (F00, F01, F02, F03), anxiety and obsessive-compulsive disorder (F40, F41, F42), substance use disorder (F10–F19).

Based on the results presented in Table 3, the SARS-CoV2 infection and COVID-19-related death risks were further examined. An additional logistic regression analysis was performed to analyse the risks according to the type of mental illness (Fig. 2). A model for estimating the SARS-CoV-2 infection risk was adjusted for health insurance premiums and CCI scores and used to calculate the risk OR for each type of mental illness. Statistical significance in SARS-CoV-2 infection risk was found according to severe mental illness (OR 2.60, 95% CI 2.21–3.06, *p*-value < 0.001), dementia (OR 1.90, 95% CI 1.62–2.22, *p*-value < 0.001) and SUD (OR 4.98, 95% CI 3.60–6.88, *p*-value < 0.001). Patients with these disorders were at a higher COVID-19 infection risk than those without.

A model for estimating the risk of COVID-19-related death was adjusted for sex, age, the region of residence, health insurance premiums and CCI scores, and was used to calculate the OR for each type of mental illness. Statistical significance in the risk of COVID-19-related death was found according to severe mental illness (OR 3.53, 95% CI 1.82–6.83, *p*-value = 0.009) and dementia (OR 2.12, 95% CI 1.39–3.22, *p*-value < 0.001). Patients with these mental illnesses were at a higher risk of dying from COVID-19 than those without.

## Discussion

In this study, we analysed SARS-CoV-2 infection and COVID-19-related death risks in patients with mental illness. Our analysis showed a higher risk of SARS-CoV-2 infection and COVID-19-related death in patients with mental illness, as well as differences according to the type of mental illness. Patients with severe mental illness or dementia were at a higher risk of SARS-CoV-2 infection and COVID-19-related death than those without. Patients with SUD were at a higher risk of SARS-CoV-2 infection and COVID-19-related death than those without. No significant difference in COVID-19-related mortality risk was found between patients with and without SUD.

A previous study analysing a national dataset from the USA found that patients with mental illness were at high risk of SARS-CoV-2 infection and those that were infected had poor COVID-19 outcomes (Wang *et al*., 2021*a*). This study analysed SARS-CoV-2 infection risk in patients with attention deficit hyperactivity disorder, bipolar disorder, depression and schizophrenia, and found that SARS-CoV-2 infection risk was high for all mental illnesses (Wang *et al*., 2021*a*). Additionally, the study found that the rate of hospitalisation due to COVID-19 was higher in patients with mental illness than in those without (Wang *et al*., 2021*a*). These results were similar to those of the present study. On the other hand, studies analysing data from the Health Insurance Review and Assessment Service (HIRA) found no significant difference in SARS-CoV-2 infection risk in patients with mental illness, but observed that COVID-19 patients with a mental illness were at a higher risk of symptom severity and death (Lee *et al*., 2020; Jeon *et al*., 2021). Nevertheless, the reason why these studies did not observe a significantly high risk of SARS-CoV-2 infection in patients with mental illness may be attributed to the fact that the infection risk for SARS-CoV-2 was compared between those with and without mental illness. In our study, we obtained more accurate results by comparing patients who did not undergo the SARS-CoV-2 test.

The high risk of SARS-CoV-2 infection in patients with mental illness can be attributed to their living environment (Adhanom Ghebreyesus, 2020). Patients with mental illness regularly visit a medical facility for outpatient treatment or reside in long-term care facilities. The WHO raised the need for infection prevention and control activities within long-term care facilities because of the high density of resident populations in these facilities (WHO, 2020*b*). Patients with mental illness may be especially susceptible to SARS-CoV-2 infection because they are protected from isolation during their socialisation therapy and come into close contact with staff members (Benson *et al*., 2020).

The significant association between severe mental illnesses (schizophrenia, bipolar disorder) and SARS-CoV2 infection risk is explained by the cognitive deterioration and consequential behavioural changes that occur in individuals with these illnesses. Patients with a severe mental illness have lower levels of education and health literacy compared to the general population (Isohanni *et al*., 2001; Tempelaar *et al*., 2017; Kim *et al*., 2019) and, thus, may lack COVID-19-related knowledge and prevention awareness. Muruganandam *et al*. conducted a questionnaire survey and interviews on COVID-19 symptoms and precautions among patients with severe mental illness who were clinically stable and found that these patients did not have sufficient COVID-19 awareness (Muruganandam *et al*., 2020). A study conducted in the UK found that the rate of SARS-CoV-2 infection was 38% (131/344) among older adults aged ⩾65 years or patients with dementia admitted to five London mental health NHS Trusts and reported that these groups were at a higher risk of infection than the general population (Livingston *et al*., 2020). Patients with dementia are at high risk of SARS-CoV-2 infection as they have difficulty maintaining a physical distance from others (Li, 2020) and their behaviour changes because of cognitive deterioration as their mental illness progresses. An American study examining COVID-19 diagnosis risk in patients with an SUD conjectured that the pharmacologic effects of drug abuse promote SARS-CoV-2 infection and increase the risk of side effects (Wang *et al*., 2021*b*). Patients with mental illness infected with SARS-CoV-2 were at a high risk of death because of their severe medical conditions and failure to receive appropriate, timely treatment. It is widely accepted that patients with mental illness have unhealthy lifestyles (Scott and Happell, 2011; Bartlem *et al*., 2015), which induces NCDs and increases the severity of SARS-CoV-2 symptoms due to complications (Hamer *et al*., 2020). Furthermore, patients with mental illness fear the social stigma around a COVID-19-positive status as they do not perceive physical symptoms and pain well (Strauss *et al*., 1990; Iancu *et al*., 2005; Stubbs *et al*., 2015) and are sensitive to stress (Chang *et al*., 2020). These characteristics of patients with mental illness cause them to postpone appropriate treatment, consequently increasing COVID-19-related mortality rates.

Patients with a severe mental illness are at a high risk of respiratory diseases such as pneumonia, which are not only caused by smoking but are also associated with antipsychotic medications (Dzahini *et al*., 2018; Haga *et al*., 2018). Respiratory diseases are comorbidities that increase the risk of COVID-19-related death (Williamson *et al*., 2020), and it may be that patients with severe mental illness are at risk of COVID-19-related death. In addition, the risk of COVID-19-related deaths in patients with underlying diseases such as cardiovascular disease (Kwenandar *et al*., 2020), Parkinson's disease (Putri *et al*., 2021) and HIV (Hariyanto *et al*., 2021) is high, which indicates that patients with mental illness with several comorbidities may have higher risk of mortality. Most patients with dementia are of advanced age and vulnerable to COVID-19-related deaths. In their systematic literature review, Hariyanto *et al*. observed high COVID-19 severity and mortality in patients with dementia and mentioned the importance of the medical management of these patients (Hariyanto *et al*., 2020).

For the reasons discussed above, patients with mental disorders should be prioritised for vaccination due to their high risk of SARS-CoV-2 infection and COVID-19-related deaths (Warren *et al*., 2020; De Picker *et al*., 2021). In addition, the patients’ caregivers should also be prioritised because of the high risk of external contact causing group infection within residential facilities for people with mental disorders (Halley and Mangurian, 2021; Woodfield *et al*., 2021).

This study had several limitations. First, we defined patients with mental illness and CCI scores based on ICD-10 codes from the health insurance claims data. However, the sensitivity of diagnoses can vary across diseases because of the nature of administrative data (Wilchesky *et al*., 2004). We tried to overcome this limitation by conservatively defining patients with mental illnesses. Second, while we investigated SARS-CoV-2 infection risk and COVID-19-related death rates after classifying mental illnesses based on major diagnosis codes, we could not account for the clinical severity of individual mental illnesses. Third, SARS-CoV-2 infection risks and COVID-19-related death rates were examined according to clinical characteristics and may have been overestimated as they were measured without separating the effect of the patients’ use of hospital services. As mentioned earlier, while patients with mental illness use hospital services, it is still necessary to measure SARS-CoV-2 infection risk and COVID-19-related death rates in community facilities. Further research on mass infection risk in long-term care facilities is needed. Despite these limitations, this study derived meaningful results regarding the risk of SARS-CoV-2 infection and COVID-19-related death using a national dataset from South Korea. We reasonably defined the control group and created a statistical model that considered the patient's financial status. We found that patients with mental illness were vulnerable to COVID-19 and presented evidence supporting the need to intervene specifically with this patient population.

In conclusion, patients with mental illness have a higher risk of SARS-CoV-2 infection and COVID-19-related death. Cognitive deterioration and living environments susceptible to SARS-CoV-2 infection increase infection rates, while severe medical conditions and delayed treatment increase COVID-19-related mortality in patients with mental illness. Patients with mental illness are a priority target for COVID-19 prevention and treatment, and it is important to plan prevention measures for this patient group.

## Data

The datasets used during the current study are available from the national health insurance service on reasonable request.

## References

[ref1] Adhanom Ghebreyesus T (2020) Addressing mental health needs: an integral part of COVID-19 response. World Psychiatry 19, 129–130. 32394569 10.1002/wps.20768 PMC7214944

[ref2] Bartlem KM, Bowman JA, Bailey JM, Freund M, Wye PM, Lecathelinais C, McElwaine KM, Campbell EM, Gillham KE and Wiggers JH (2015) Chronic disease health risk behaviours amongst people with a mental illness. Australian & New Zealand Journal of Psychiatry 49, 731–741. 10.1177/0004867415569798 25698807

[ref3] Benson NM, Öngür D and Hsu J (2020) COVID-19 testing and patients in mental health facilities. The Lancet Psychiatry 7, 476–477. 32407671 10.1016/S2215-0366(20)30198-X PMC7213967

[ref4] Chang KC, Strong C, Pakpour AH, Griffiths MD and Lin CY (2020) Factors related to preventive COVID-19 infection behaviors among people with mental illness. Journal of the Formosan Medical Association 119, 1772–1780. 32773260 10.1016/j.jfma.2020.07.032 PMC7388748

[ref5] De Picker LJ, Dias MC, Benros ME, Vai B, Branchi I, Benedetti F, Borsini A, Leza JC, Kärkkäinen H, Männikkö M, Pariante CM, Sönmez Güngör E, Szczegielniak A, Tamouza R, van der Markt A, Fusar-Poli P, Beezhold J and Leboyer M (2021) Severe mental illness and European COVID-19 vaccination strategies. Lancet Psychiatry 8, 356–359. 33609450 10.1016/S2215-0366(21)00046-8 PMC7906735

[ref6] Doung-Ngern P, Suphanchaimat R, Panjangampatthana A, Janekrongtham C, Ruampoom D, Daochaeng N, Eungkanit N, Pisitpayat N, Srisong N, Yasopa O, Plernprom P, Promduangsi P, Kumphon P, Suangtho P, Watakulsin P, Chaiya S, Kripattanapong S, Chantian T, Bloss E, Namwat C and Limmathurotsakul D (2020) Case-control study of use of personal protective measures and risk for SARS-CoV 2 infection, Thailand. Emerging Infectious Diseases 26, 2607–2616. 32931726 10.3201/eid2611.203003 PMC7588529

[ref7] Druss BG (2020) Addressing the COVID-19 pandemic in populations with serious mental illness. JAMA Psychiatry 77, 891–892. 32242888 10.1001/jamapsychiatry.2020.0894

[ref8] Dzahini O, Singh N, Taylor D and Haddad PM (2018) Antipsychotic drug use and pneumonia: systematic review and meta-analysis. Journal of Psychopharmacology 32, 1167–1181. 30334664 10.1177/0269881118795333

[ref9] Haga T, Ito K, Sakashita K, Iguchi M, Ono M and Tatsumi K (2018) Risk factors for pneumonia in patients with schizophrenia. Neuropsychopharmacology Reports 38, 204–209. 30353691 10.1002/npr2.12034 PMC7292272

[ref10] Halley MC and Mangurian C (2021) Caring for the caregivers – Covid-19 vaccination for essential members of the health care team. New England Journal of Medicine 384, e33. 10.1056/NEJMpv2101339 PMC8721589 33577149

[ref11] Hamer M, Kivimaki M, Gale CR and Batty GD (2020) Lifestyle risk factors, inflammatory mechanisms, and COVID-19 hospitalization: a community-based cohort study of 387,109 adults in UK. Brain, Behavior, and Immunity 87, 184–187. 10.1016/j.bbi.2020.05.059 PMC7245300 32454138

[ref12] Hariyanto TI, Putri C, Arisa J, Situmeang RFV and Kurniawan A (2020) Dementia and outcomes from coronavirus disease 2019 (COVID-19) pneumonia: a systematic review and meta-analysis. Archives of Gerontology and Geriatrics 93, 104299. 33285424 10.1016/j.archger.2020.104299 PMC7674980

[ref13] Hariyanto TI, Rosalind J, Christian K and Kurniawan A (2021) Human immunodeficiency virus and mortality from coronavirus disease 2019: a systematic review and meta-analysis. Southern African Journal of HIV Medicine 22, a1220. 10.4102/sajhivmed.v22i1.1220 PMC8063497 33936793

[ref14] Himelhoch S, Lehman A, Kreyenbuhl J, Daumit G, Brown C and Dixon L (2004) Prevalence of chronic obstructive pulmonary disease among those with serious mental illness. American Journal of Psychiatry 161, 2317–2319. 10.1176/appi.ajp.161.12.2317 15569908

[ref15] Iancu I, Strous R, Poreh A, Kotler M and Chelben Y (2005) Psychiatric inpatients’ reactions to the SARS epidemic: an Israeli survey. Israel Journal of Psychiatry and Related Sciences 42, 258. 16618059

[ref16] Isohanni I, Jones PB, Järvelin M-R, Nieminen P, Rantakallio P, Jokelainen J, Croudace TJ and Isohanni M (2001) Educational consequences of mental disorders treated in hospital. A 31-year follow-up of the Northern Finland 1966 Birth Cohort. Psychological Medicine 31, 339. 11232920 10.1017/s003329170100304x

[ref17] Jeon HL, Kwon JS, Park SH and Shin JY (2021) Association of mental disorders with SARS-CoV-2 infection and severe health outcomes: nationwide cohort study. British Journal of Psychiatry 218, 344–351. 10.1192/bjp.2020.251 33407954

[ref18] Kim SW, Park WY, Jhon M, Kim M, Lee JY, Kim SY, Kim JM, Shin IS and Yoon JS (2019) Physical health literacy and health-related behaviors in patients with psychosis. Clinical Psychopharmacology and Neuroscience 17, 279. 30905128 10.9758/cpn.2019.17.2.279 PMC6478081

[ref19] Kwenandar F, Japar KV, Damay V, Hariyanto TI, Tanaka M, Lugito NPH and Kurniawan A (2020) Coronavirus disease 2019 and cardiovascular system: a narrative review. IJC Heart & Vasculature 29, 100557. 32550259 10.1016/j.ijcha.2020.100557 PMC7266760

[ref20] Lee SW, Yang JM, Moon SY, Yoo IK, Ha EK, Kim SY, Park UM, Choi S, Lee SH, Ahn YM, Kim JM, Koh HY and Yon DK (2020) Association between mental illness and COVID-19 susceptibility and clinical outcomes in South Korea: a nationwide cohort study. Lancet Psychiatry 7, 1025–1031. 32950066 10.1016/S2215-0366(20)30421-1 PMC7498216

[ref21] Li L (2020) Challenges and priorities in responding to COVID-19 in inpatient psychiatry. Psychiatric Services 71, 624–626. 32321388 10.1176/appi.ps.202000166

[ref22] Livingston G, Rostamipour H, Gallagher P, Kalafatis C, Shastri A, Huzzey L, Liu K, Sommerlad A and Marston L (2020) Prevalence, management, and outcomes of SARS-CoV-2 infections in older people and those with dementia in mental health wards in London, UK: a retrospective observational study. Lancet Psychiatry 7, 1054–1063. 33031760 10.1016/S2215-0366(20)30434-X PMC7535621

[ref23] Ministry of Health and Welfare (2020) Coronavirus Disease-19, Republic of Korea. Available at http://ncov.mohw.go.kr/ (Accessed 1 January 2020).

[ref24] Momen NC, Plana-Ripoll O, Agerbo E, Benros ME, Børglum AD, Christensen MK, Dalsgaard S, Degenhardt L, Jonge P, Debost JC, Fenger-Grøn M, Gunn JM, Iburg KM, Kessing LV, Kessler RC, Laursen TM, Lim C, Mors O, Mortensen PB, Musliner KL, Nordentoft M, Pedersen CB, Petersen LV, Ribe AR, Roest AM, Saha S, Schork AJ, Scott KM, Sievert C, Sørensen HJ, Terry J, Stedman TJ, Vestergaard M, Vilhjalmsson B, Werge T, Weye N, Whiteford HA, Prior A and McGrath JJ (2020) Association between mental disorders and subsequent medical conditions. New England Journal of Medicine 382, 1721–1731. 10.1056/NEJMoa1915784 PMC7261506 32348643

[ref25] Muruganandam P, Neelamegam S, Menon V, Alexander J and Chaturvedic SK (2020) COVID-19 and severe mental illness: impact on patients and its relation with their awareness about COVID-19. Psychiatry Research 291, 113265. 32763536 10.1016/j.psychres.2020.113265 PMC7322460

[ref26] National Health Insurance Service (2020) National Health Insurance Sharing Service. Available at https://nhiss.nhis.or.kr/bd/ay/bdaya001iv.do (Accessed 26 December 2020).

[ref27] Putri C, Hariyanto TI, Hananto JE, Christian K, Situmeang RFV and Kurniawan A (2021) Parkinson's disease may worsen outcomes from coronavirus disease 2019 (COVID-19) pneumonia in hospitalized patients: a systematic review, meta-analysis, and meta-regression. Parkinsonism & Related Disorders 87, 155–161. 33931304 10.1016/j.parkreldis.2021.04.019 PMC8065236

[ref28] Quan H, Li B, Couris CM, Fushimi K, Graham P, Hider P, Januel JM and Sundararajan V (2011) Updating and validating the Charlson comorbidity index and score for risk adjustment in hospital discharge abstracts using data from 6 countries. American Journal of Epidemiology 173, 676–682. 21330339 10.1093/aje/kwq433

[ref29] Richardson S, Hirsch JS, Narasimhan M, Crawford JM, McGinn T, Davidson KW and the Northwell COVID-19 Research Consortium (2020) Presenting characteristics, comorbidities, and outcomes among 5700 patients hospitalized with COVID-19 in the New York City area. JAMA 323, 2052–2059. 32320003 10.1001/jama.2020.6775 PMC7177629

[ref30] Scott D and Happell B (2011) The high prevalence of poor physical health and unhealthy lifestyle behaviours in individuals with severe mental illness. Issues in Mental Health Nursing 32, 589–597. 21859410 10.3109/01612840.2011.569846

[ref31] Shi Q, Hu Y, Peng B, Tang XJ, Wang W, Su K, Luo C, Wu B, Zhang F, Zhang Y, Anderson B, Zhong XN, Qiu JF, Yang CY and Huang AL (2021) Effective control of SARS-CoV-2 transmission in Wanzhou, China. Nature Medicine 27, 86–93. 10.1038/s41591-020-01178-5 33257893

[ref32] Shinn AK and Viron M (2020) Perspectives on the COVID-19 pandemic and individuals with serious mental illness. Journal of Clinical Psychiatry 81, 20com13412. 10.4088/JCP.20com13412 32369691

[ref33] Strauss DH, Spitzer RL and Muskin PR (1990) Maladaptive denial of physical illness: a proposal for DSM-IV. American Journal of Psychiatry 147, 1168–1172. 10.1176/ajp.147.9.1168 2133370

[ref34] Stubbs B, Thompson T, Acaster S, Vancampfort D, Gaughran F and Correll CU (2015) Decreased pain sensitivity among people with schizophrenia: a meta-analysis of experimental pain induction studies. Pain 156, 2121–2131. 26207650 10.1097/j.pain.0000000000000304

[ref35] Tempelaar WM, Termorshuizen F, MacCabe JH, Boks MP and Kahn RS (2017) Educational achievement in psychiatric patients and their siblings: a register-based study in 30 000 individuals in the Netherlands. Psychological Medicine 47, 776–784. 27873559 10.1017/S0033291716002877

[ref36] Walker ER, McGee RE and Druss BG (2015) Mortality in mental disorders and global disease burden implications: a systematic review and meta-analysis. JAMA Psychiatry 72, 334–341. 25671328 10.1001/jamapsychiatry.2014.2502 PMC4461039

[ref37] Wang Q, Xu R and Volkow ND (2021 *a*) Increased risk of COVID-19 infection and mortality in people with mental disorders: analysis from electronic health records in the United States. World Psychiatry 20, 124–130. 33026219 10.1002/wps.20806 PMC7675495

[ref38] Wang QQ, Kaelber DC, Xu R and Volkow ND (2021 *b*) COVID-19 risk and outcomes in patients with substance use disorders: analyses from electronic health records in the United States. Molecular Psychiatry 26, 30–39. 32929211 10.1038/s41380-020-00880-7 PMC7488216

[ref39] Warren N, Kisely S and Siskind D (2020) Maximizing the uptake of a COVID-19 vaccine in people with severe mental illness: a public health priority. JAMA Psychiatry 78, 589–590. 10.1001/jamapsychiatry.2020.4396 33320243

[ref40] WHO (2020 *a*) *COVID-19 dashboard*. Available at https://covid19.who.int/ (Accessed 1 January 2020).

[ref41] WHO (2020 *b*) Infection prevention and control guidance for long-term care facilities in the context of COVID-19: interim guidance. Available at https://apps.who.int/iris/handle/10665/331508 (Accessed 21 March 2020).

[ref42] Wiersinga WJ, Rhodes A, Cheng AC, Peacock SJ and Prescott HC (2020) Pathophysiology, transmission, diagnosis, and treatment of coronavirus disease 2019 (COVID-19): a review. JAMA 324, 782–793. 32648899 10.1001/jama.2020.12839

[ref43] Wilchesky M, Tamblyn RM and Huang A (2004) Validation of diagnostic codes within medical services claims. Journal of Clinical Epidemiology 57, 131–141. 15125622 10.1016/S0895-4356(03)00246-4

[ref44] Williamson EJ, Walker AJ, Bhaskaran K, Bacon S, Bates C, Morton CE, Curtis HJ, Mehrkar A, Evans D, Inglesby P, Cockburn J, McDonald HI, MacKenna B, Tomlinson L, Douglas IJ, Rentsch CT, Mathur R, Wong AY, Grieve R, Harrison D, Forbes H, Schultze A, Croker R, Parry J, Hester F, Harper S, Perera R, Evans SJ, Smeeth L and Goldacre B (2020) Factors associated with COVID-19-related death using OpenSAFELY. Nature 584, 430–436. 32640463 10.1038/s41586-020-2521-4 PMC7611074

[ref45] Woodfield MC, Pergam SA and Shah PD (2021) Cocooning against COVID-19: the argument for vaccinating caregivers of patients with cancer. American Cancer Society Journals 127, 2861–2863. 10.1002/cncr.33598 PMC8251451 33891713

[ref46] Yao H, Chen J-H and Xu Y-F (2020) Patients with mental health disorders in the COVID-19 epidemic. Lancet Psychiatry 7, e21. 32199510 10.1016/S2215-0366(20)30090-0 PMC7269717

[ref47] Zhou F, Yu T, Du R, Fan G, Liu Y, Liu Z, Xiang J, Wang Y, Song B, Gu X, Guan L, Wei Y, Li H, Wu X, Xu J, Tu S, Zhang Y, Chen H and Cao B (2020) Clinical course and risk factors for mortality of adult inpatients with COVID-19 in Wuhan, China: a retrospective cohort study. The Lancet 395, 1054–1062. 10.1016/S0140-6736(20)30566-3 PMC7270627 32171076

